# The Influence of Leader-Follower Cognitive Style Similarity on Followers’ Organizational Citizenship Behaviors

**DOI:** 10.3389/fpsyg.2020.01265

**Published:** 2020-06-09

**Authors:** Steven J. Armstrong, Meng Qi

**Affiliations:** ^1^College of Economics & Management, Beijing University of Technology, Beijing, China; ^2^Lincoln International Business School, University of Lincoln, Lincoln, United Kingdom

**Keywords:** organizational citizenship behavior, cognitive style, congruence, polynomial regression, response surface analysis

## Abstract

While cognitive style congruence has been highlighted as a potentially important variable influencing performance outcomes in work-related contexts, studies of its influence are scarce. This paper examines the influence of leader-follower cognitive style similarity on followers’ organizational citizenship behaviors (OCBs). Data from 430 leader-follower dyads were analyzed using polynomial regression and response surface analysis. Results demonstrate that congruence of leader/follower cognitive style is a predictor of follower OCBs. Organizations may therefore benefit from considering issues of similarity of cognitive styles in their attempts to develop effective leader-follower partnerships leading to increased OCBs and concomitant improvements in both individual and organizational level success.

## Introduction

Organizations that rely solely on job roles to elicit work related behaviors are at a distinct disadvantage compared with those that focus on eliciting extra role behaviors (Bowler and Brass, 2006). Such assertions underpin the importance of a significant body of research known as organizational citizenship behavior (OCB) that is concerned with harnessing both social and intellectual capital of employees (Podsakoff et al., 2014). OCBs have emerged as one of the most important constructs in the fields of Human Resource Management (Snape and Redman, 2010) and Organizational Psychology (Miao et al., 2017).

Since previous research has revealed that OCBs contribute to significant improvements in organizational-level performance and success (Podsakoff et al., 2009), it is important to understand the factors that lead employees to perform these behaviors. Previous research in this area has focused on four major categories of antecedents of OCB: individual characteristics; task characteristics; organizational characteristics; and leadership behaviors (Ernhart, 2004). Our study seeks to extend the body of literature associated with the former. Most previous research in this category has focused on individual-level predictors of OCB such as personality, employee attitudes, employee role perceptions, employee abilities, and dispositional variables (Son and Kim, 2016). However, as Chung et al. (2011) remind us, “OCBs are performed not by isolated actors but by people in formal and informal social structures” (Lamertz, 2005, p. 2) where social relationships and diversity of organizational members have been found to exert significant influences on employees’ helping behaviors (Bowler and Brass, 2006; Venkataramani and Dalal, 2007).

From this social perspective, differences among members in the workplace can lead to a source of us-and-them distinctions resulting in a negative influence on social integration, reduced cooperation and cohesion, and decreased performance of group members (O’Reilly et al., 1989; Harrison et al., 1998). Conversely, similarity among members is known to result in more of a willingness to collaborate, leading to smoother interactions (McGrath, 1984), increased friendship (Antill, 1984), and higher levels of group cohesion (Tsui and O’Reilly, 1989). The conceptual foundation that renders homogeneity as being more conducive to group performance than diversity (Bell and Villado, 2011) has led to a variety of similarity-attraction theories emerging from the fields of organizational behavior (e.g., Schneider, 1987; Milliken and Martins, 1996) and social psychology (e.g., Byrne, 1971; McGrath, 1984).

Early studies of diversity from an individual differences perspective (e.g., Pfeffer, 1983) focused on overt demographic differences among employees such as heterogeneity in age, gender, ethnicity and organizational tenure. However, effects of heterogeneity using these surface-level variables were inconsistent and weaker than expected. This led to a call for more studies of deep-level diversity involving underlying attributes that cannot be easily detected such as differences among members’ knowledge, skills, values, beliefs and attitudes (Jackson et al., 1995; Milliken and Martins, 1996). Deep-level diversity of this nature becomes apparent only after interaction with the particular person, and has been shown to be particularly problematic for work-group cohesion (Harrison et al., 1998). Our study responds to recent calls for more research into these deep-level differences (van Knippenberg and Schippers, 2007), especially in the context of cognitive diversity (Martins et al., 2012).

Despite its high relevance, the growing body of literature on cognitive diversity has been criticized for suffering from varied conceptual and operational definitions that restrict theory development and comparisons of empirical results. In response, Mello and Rentsch (2015) provide guidance for the systematic study of cognitive diversity and team functioning by offering an organizing heuristic (of the literature) based on four levels of stability associated with the cognitive diversity conceptualization. These are: *trait-like*; *developmental*; *acquired*; *exposed* and were put forward to delineate the effects of cognitive diversity on performance. Of these, *trait-like* represents the most stable cognitive variables that are innate characteristics of the individual. Examples include personality, information processing styles, cognitive ability, and cognitive styles. According to Mello and Rentsch (2015), “trait-like cognitive diversity has broad explanatory power” (p. 638) and, in particular, “cognitive style research yields the most consistent results, but overall there is much more work needed to draw solid conclusions” (Mello and Rentsch, 2015). We extend this line of inquiry by examining the possibility that congruence of cognitive style between leaders and their followers in the workplace may result in improved interpersonal relationships, and, on the basis of social exchange theory, concomitant positive influences on follower OCB.

Our study provides a number of important contributions to the literature. First, we examine the relationship between OCBs and deep-level leader-subordinate relations for which there are a dearth of previous studies (Matta and van Dyne, 2015). Second, we extend the literature on deep-level workplace diversity by incorporating theory from a growing body of research into cognitive diversity (Martins et al., 2012). Third, whilst most previous studies of OCB have focused on the perspective of either the follower or the leader (Muldoon et al., 2017), ours considers the role of individual differences and interactions between leaders and their followers in the production of citizenship behaviors. Finally, whilst the influence of cognitive style similarity has been studied in a number of different contexts (Armstrong et al., 2012), its influence on OCBs has never been examined.

As importantly, our study seeks to provide a number of important contributions to the business world. Given that OCBs are widely recognized as being critical in organizations where performance, flexibility, knowledge sharing, and the development of social capital to underpin long-term success are important, our study seeks to provide practical ways in which these bahaviors can be maximized. Our article sets out to demonstrate that this can be achieved by demonstrating that: cognitive similarity in leader-subordinate dyads is a critically important underpinning variable; cognitive style awareness needs to feature in leadership development programs and recruitment and selection strategies; appropriate matching at the leader-subordinate dyadic level is crucial. Through these means, we expect that enhanced levels of OCBs will contribute to organizational effectiveness and therefore have a noticeable impact on the success and welfare of individuals and on financial measures of an organization’s success.

## Theory Development and Hypotheses

### Organizational Citizenship Behaviors (OCBs)

Defined as “individual behavior that is discretionary, not directly or explicitly recognized by the formal reward system, and that in aggregate promotes the effective functioning of the organization” (Organ, 1988, p. 4), OCB has emerged as one of the most important constructs in organizational psychology (Miao et al., 2017) and has a sizeable impact on the welfare and success of both individuals and organizations (Chin, 2015). Organ (1988) put forward a 5-factor model of OCB based on five types of citizenship behavior referred to as: sportsmanship, civic virtue, conscientiousness, altruism and courtesy. These five factors were later defined by Podsakoff et al’s (1990, p. 115) as follows:

*Sportsmanship:* willingness of the employee to tolerate less than ideal circumstances without complaining.
*Conscientiousness*: discretionary behaviors on the part of the employee that go well beyond the minimum role requirements of the organization, in the areas of attendance, obeying rules and regulations, taking breaks, and so forth.
*Civic virtue:* behaviors that indicate employees take an active interest in the life of their organization.
*Altruism:* discretionary behaviors that have the effect of helping a specific other person with an organizationally relevant task or problem.
*Courtesy:* discretionary behavior on the part of an individual aimed at preventing work-related problems with others from occurring.

Managers have little difficulty in distinguishing between the *Sportsmanship, Conscientiousness*, and *Civic virtue* factors in terms of their consequences for the organization (Hui et al., 2004). However, difficulties are experienced in making distinctions between the dimensions of *Altruism/Courtesy* and consequences for the organization because these tend to be viewed as part of an overall helping dimension (Bachrach et al., 2001). This led to a categorization on the basis of a two-dimensional structure of OCB determined by the direction or target of the behaviors. Drawing on Williams and Anderson’s (1991) earlier work, Podsakoff et al. (2014) referred to those behaviors directed toward helping other individuals that indirectly contribute to the organization (*Altruism* and *Courtesy*) as OCBI, and those behaviors directed toward the specific benefit of the organization (*Sportsmanship, Conscientiousness*, and *Civic virtue*) as OCBO.

Previous findings have revealed that overall OCBs are positively related to organizational effectiveness measures such as profitability, efficiency and productivity, as well as individual level effectiveness measures such as employee performance, appraisal ratings, and reward allocation decisions (Podsakoff et al., 2009). In view of these findings, it is important that we continue in our quest to more fully understand the factors that lead employees to perform these behaviors. Of the four major categories of previous research on OCB antecedents identified by Ernhart (2004) as individual characteristics, task characteristics, organizational characteristics and leadership behaviors, our study seeks to extend the body of literature associated with the former. More specifically, we respond to calls for more studies that examine deep-level cognitive differences (van Knippenberg and Schippers, 2007) between individuals in the context of workplace diversity.

### Workplace Diversity

Workplace diversity is reported to lead to problems with coordination and communication (Jackson et al., 1995), negative effects on achieving strategic consensus (Aggarwal and Woolley, 2013), and negative consequences for affective reactions such as cohesion, satisfaction, and commitment (Jackson et al., 2003). The two main traditions of research into work-group diversity have been identified as the *social categorization perspective* and the *information/decision making perspective* (Williams and O’Reilly, 1998). The latter points to the positive effects of diversity on the basis that individual differences will inspire flexible and divergent thinking that enables new patterns of thought and more creative outcomes (Homan et al., 2015). Differences may also be associated with valuable task relevant knowledge and expertise which expands the available information (Pieterse et al., 2011) and leads to conflicting viewpoints on the task at hand resulting in more thorough processing of task-based information (van Knippenberg and Schippers, 2007).

In contrast, the *social categorization perspective*, upon which the present study is focused, regards diversity as a source of us-and-them distinctions where dissimilar others are seen as belonging to an out-group leading to decreased cohesion, coordination, and cooperation among team members that ultimately leads to decreased performance (Milliken and Martins, 1996). This perspective draws on Byrne’s (1997) similarity-attraction theory which suggests that individuals are more attracted to similar others. Consequently, members are more willing to collaborate with others similar to themselves resulting in smoother interactions and thus rendering homogeneity more conducive to group performance than diversity (Bell and Villado, 2011). This in-group/out-group distinction leads to members developing intergroup bias and in some circumstances to cooperate with, and favor in-group members more than out-group members (van Knippenberg et al., 2004).

#### Deep-Level Cognitive Diversity

It will be recalled that deep-level diversity (e.g., skills, values, beliefs) becomes apparent only after interaction with the particular person, and can be problematic for work-group cohesion (Harrison et al., 1998). Our study responds to calls for more research into deep-level differences (van Knippenberg and Schippers, 2007), particularly those related to cognitive diversity (Martins et al., 2012). Our focus is at the dyad level (i.e., leader-follower) rather than teams which has been the focus of most previous research, although the degree to which members are psychologically linked or attracted toward interacting with one another in pursuit of a common objective are likely to be no different (Milliken and Martins, 1996; Tsui et al., 2002). Indeed, basic processes such as potential for conflict and collaboration, influence attempts, and face-face communication characterize both teams and dyads alike (Harrison et al., 1998).

Our thinking is based on the social categorization perspective of diversity (Williams and O’Reilly, 1998) and draws on the similarity-attraction paradigm (Byrne, 1971) whose effects on interpersonal interactions are one of the most robust phenomena in social psychology (Davendorf and Highhouse, 2008). The theory posits that dissimilarity in personal attributes tends to engender repulsion, whereas individuals are attracted to, and like others who are similar to themselves (Byrne, 1997). The similarity effect has been observed in a variety of situations and remains robust when set alongside a number of determining factors such as personality traits, attitudes, demographics and even physical attractiveness (Montoya and Horton, 2004). In a work context it has been shown that followers who regard themselves as being similar to their supervisors are rated as being higher performers than others (Turban and Jones, 1988). Perceived similarity among leader-follower dyads also leads to increased liking (Turban et al., 1990), mutual trust and respect (Dienesch and Liden, 1986) and increased levels of rapport resulting in higher levels of interaction and higher quality exchange relationships (Deluga, 1998). Follower satisfaction also increases due to leaders increasing both tangible (e.g., career advancement) and intangible benefits such as having a trust-based relationship (Erdogan and Enders, 2007). Conversely, there is evidence to suggest that supervisors tend to perceive dissimilar followers less positively and tend to give them lower performance ratings (Milliken and Martins, 1996).

According to social exchange theory (Blau, 1964), when followers observe that they receive support, trust, and other tangible and intangible benefits from their leaders they feel more satisfied (Newman et al., 2016) and feel obliged to reciprocate the positive treatment they have been granted by engaging in behavior that directly benefits the organization (Chin, 2015), including OCBs (Kabasakal et al., 2011).

### Cognitive Style Congruence

It will be recalled that the majority of previous studies of OCB have focused on individual-level predictors such as personality, trust, equity and relationship quality (Son and Kim, 2016). However, a growing area of interest in the field of workplace diversity has revealed that trait-like cognitive diversity has broad explanatory power (Mello and Delise, 2015) and that in particular “cognitive style research yields the most consistent results, but overall there is more work needed to draw solid conclusions” (Mello and Rentsch, 2015, p. 638). We seek to extend this line of inquiry within the context of OCB research. Cognitive style has been defined as consistent individual differences in how individuals perceive, think, process information, solve problems, learn, take decisions and relate to others (Armstrong et al., 2012). A number of variables relevant to interpersonal relationships have been examined in relation to congruence between cognitive styles of individuals interacting with each other. For example congruent cognitive styles have been found to be associated with: satisfaction with the relationship (Cooper and Miller, 1991); effective interpersonal relations (Handley, 1982); mutually positive attitudes between parties in a relationship (Renninger and Snyder, 1983); and mutual understanding and liking (Myers, 1980). More recently, Suazo et al. (2008) observed that “congruence of cognitive styles should result in increased levels of interpersonal attraction, greater communication, and reduced ambiguity in the leader-subordinate dyad” (p. 3). One prominent cognitive style dimension that has been shown to fundamentally affect the nature of interpersonal relationships in this way is the intuitive-analytic dimension (Armstrong, 1999).

#### Intuitive-Analytic Cognitive Styles

Due to the absence of a valid and reliable instrument suitable for use in large-scale management and organizational studies, Allinson and Hayes (1996) developed the Cognitive Style Index (CSI) for assessing individuals’ positions on the generic intuition-analysis dimension of cognitive style (Agor, 1984; Hammond et al., 1987; Simon, 1987). The CSI is a self-report, bi-polar, unidimensional questionnaire that measures individuals’ cognitive styles on a range from highly intuitive to highly analytic. Intuition refers to immediate judgment based on feeling and the adoption of a global perspective. People with this cognitive style work best on unstructured problems. They prefer rapid and open-ended approaches to decision making, relying on random methods of exploration based on immediate judgment and feeling (Lynch, 1986). People with this style tend to adopt an “interpersonal” approach to problem solving (Armstrong et al., 2004). Conversely, analysis refers to judgment based on mental reasoning and a focus on detail. Analytic individuals prefer a more structured approach to decision making, applying systematic methods of investigation using mental reasoning. They prefer to work on problems requiring a step-by-step solution and tend to adopt an “impersonal” approach to problem solving (Pascual-Leone, 1989).

At this point we should note that there is some controversy over two incompatible perspectives on the relationship between intuition and analysis. This concerns the distinction between whether intuition and analysis are opposite poles of a single dimension (unitary perspective) or whether they are orthogonal constructs (complex perspective). For example, Wang et al. (2017) conducted a meta-analytic study of the relation between intuition and analysis and concluded that these are independent constructs. However, their analyses were based on a range of instruments that were designed to specifically assess intuition and analysis separately. It is unsurprising, therefore, that they found the two constructs to be uncorrelated. Other studies in the field of cognitive science express grave reservations for the existence of two distinct cognitive architectures. Keren and Schul (2009) offered a particularly detailed critique of the dual-systems theories concluding that, contrary to the dualistic premises, dimensions assumed to distinguish the two systems (e.g., intuitive versus analytic) are continuous rather than dichotomous. Kahneman (2011) also described dual cognitive systems as “useful fictions” that help us explain quirks in decision making. On the basis of Keren and Schul’s (2009) earlier work, Kruglanski and Gigerenzer (2011) provided convergent arguments and evidence for a unified theoretical approach to intuitive and analytic judgments.

These debates over the nature of intuition-analysis being a unitary or complex phenomenon have also been leveled at the construct validity of the CSI. For example, Hodgkinson and Sadler-Smith (2003) assert that the uni-dimensional conception of the CSI adopted by Allinson and Hayes (1996) downplays the extant literature that depicts a picture of higher complexity. They also provided some empirical evidence suggesting that a two-factor model provides a better approximation of responses to the CSI. In their rebuttal, Hayes et al. (2003) concluded that these authors had failed to present a robust challenge to the construct validity of the CSI. Allinson and Hayes (2012) later asserted that “to regard intuition and analysis as independent dimensions would be to deny a centuries-old perception of individual thought processes that can be traced back at least to the writings of Aristotle, as well as sacrificing the most parsimonious explanation of cognitive style” (p. 3). Further studies were undertaken in an attempt to either replicate or refute Hodgkinson and Sadler-Smith (2003) earlier assertions. These studies (Hammad, 2012; Armstrong and Qi, 2016; Cuneo, 2020) reported findings of a series of confirmatory factor analyses suggesting that research using the CSI should continue on the basis of its original uni-factorial structure.

#### Dyadic Influences of Cognitive Style Diversity

In terms of dyadic influences, cognitive style diversity is based on the premise that members are likely to have different cognitive styles. That is, in a given dyad, individual members are likely to occupy different positions on the continuum that runs from a strong preference for an intuitive orientation to a strong preference for an analytic orientation. The degree of difference in cognitive style between members within a given dyad will determine the extent to which that dyad is homogenous or heterogeneous – e.g., its level of congruence/diversity. In a work context an analytic person would tend to focus on hard data, breaking problems down into their constituent parts, and studying each part in detail. They tend to adopt a systematic search for understanding via a logical step-by-step analysis and take an impersonal and structured approach to decision making. Conversely, an intuitive person would be more receptive to soft data, often experiencing an immediate sense of knowing which they cannot explain, and adopt a more global approach to processing information. They tend to emphasize synthesis and the simultaneous integration of many inputs at the same time, and prefer a more open, interpersonal and rapid approach to decision making using random methods of exploration (Armstrong et al., 2012).

Whilst the influence of cognitive style similarity in dyads working within organizations has been examined in a number of different contexts (e.g., Armstrong, 1999; Allinson et al., 2001; Armstrong et al., 2002; Vanderheyden and De-Baets, 2015) its direct influence on OCBs has never been examined. Although previous findings are mixed, there is evidence to suggest that cognitive style congruence not only enhances the quality of dyadic relationships, but also works indirectly through its influence on other variables to enhance mutual understanding and liking (Myers, 1980) and other behavioral and attitudinal manifestations such as trust, admiration, empathy and respect (Armstrong et al., 2002). Studies have also shown that similarities in cognitive style result in reduced ambiguity, increased levels of interpersonal attraction, and better communication in leader-follower dyads (Johlke and Duhan, 2001), resulting in fewer misunderstandings and enhanced leader-follower relationships (Suazo et al., 2008). Conversely, dissimilarities in cognitive styles accentuate the negative characteristics of a dyadic relationship (Tsui et al., 2002) and can often result in conflict (Leonard and Straus, 1997). This is unsurprising since it is known that people who are highly analytical do not readily combine with those who are highly intuitive – they often tend to be irritated by, and hold pejorative views of each other (Kirton, 1989). Furthermore, it is known that leaders tend to perceive dissimilar followers less positively and tend to give them lower performance ratings (Milliken and Martins, 1996).

It is clear then, that differences in cognitive style fundamentally affect interpersonal relationships and that interaction between people should proceed more harmoniously, when, “as a function of similarity in style, they perceive and process information in similar ways, and use similar modes of communication” (Armstrong et al., 2012, p. 244). The degree of harmony an employee perceives is known to be positively related to employees’ displaying OCBs reciprocally toward the organization (Chiu and Chen, 2005; Kabasakal et al., 2011; Chin, 2015). This leads us to our first hypothesis:

*H*_1_: Leader-follower cognitive style similarity positively predicts followers’ overall organizational citizenship behaviors.

### Dimensionality of OCBs

With regard to dimensionality of OCBs, consequences were categorized by Williams and Anderson (1991) on the basis of the direction of behavior toward either the benefit of individuals (OCBI) or toward the benefit of the organization (OCBO). According to Podsakoff et al. (2009), factors associated with OCBI include: *Courtesy –* helping others to solve problems; and *Altruism –* voluntary behaviors to help other people in the organization. Helping in this context is a type of interpersonal, cooperative, and affiliative extra-role behavior directed toward members of one’s workgroup (Van Dyne and LePine, 1998). These behaviors occur without any external rewards and do not have punitive consequences when not performed by the employee (Liao et al., 2008). Such behaviors have been shown to result from good quality interpersonal relationships that promote mutual concern and increased sensitivity to the needs of others (McAllister, 1995). Leaders who recognize interpersonal citizenship behaviors in their followers such as altruism, courtesy (Hoffman et al., 2007) and other helping behaviors are likely to reciprocate (Homans, 1961) through increased liking and trust in those employees (Dienesch and Liden, 1986). This has been found to positively influence leaders’ performance evaluations and reward distribution (Lefkowitz, 2000) that subsequently leads to reinforcement of subordinates work-role behaviors and increased job satisfaction (Erdogan and Enders, 2007).

These helping behaviors associated with OCBIs are characteristic of those behaviors associated with people whose cognitive styles are more intuitive than analytic. For example, intuitive individuals are known to have a social orientation and encompass a strong interest in people with a preference for being with and helping others – e.g., *Courtesy*– (Witkin and Goodenough, 1977; Armstrong et al., 2002). Intuitive people also tend to promote effective functioning in workplace settings by maintaining positive interpersonal relationships – e.g., *Altruism* – and exhibiting warm and nurturing behavior (Armstrong, 1999). They are also more likely to shift their opinions to resolve conflicts while analytic people tend to be less willing to adapt their views to those of others (Armstrong et al., 2002).

Similarities in vertical dyads have revealed consistent and lasting positive effects on supervisor related performance, relationship quality, and the promotion opportunities of subordinates (Deluga, 1998), whereas dissimilarity leads to less favorable job attitudes and a lower willingness to help others (Schaubroeck and Lam, 2002). Suazo et al. (2008) revealed that similarity in cognitive style in particular is associated with higher quality leader-subordinate relations. A later study of the analytic-intuitive dimension of cognitive style revealed that whilst congruence increases communication satisfaction between leaders and their subordinates, this was significantly higher when leaders and their subordinates were intuitive rather than analytic (Erdil and Tanova, 2015). Other studies (e.g., Liao et al., 2008) of the effect of deep-level similarity also revealed that working partners will be more committed and more satisfied with job experiences within a work-group and will more willingly engage in cooperative helping behaviors toward co-workers. Deep-level leader-subordinate similarity has also been shown by Huang and Iun (2006) to have significant effects on extra-role performance using Lee and Allen’s (2002) OCB scale.

On the basis that OCBIs are about helping others within organizations through cooperative and affiliative extra-role behaviors, that such behaviors are more reflective of individuals with intuitive rather than analytic cognitive styles, and that deep level similarities in vertical dyads are known to lead to a greater willingness on the part of subordinates to engage in these sorts of behaviors, we hypothesize that:

*H*_2_: Leader-follower congruence at the extreme intuitive end of the cognitive style continuum (intuitive follower-intuitive leader) will lead to higher levels of follower OCBIs being reported by their leaders.

According to Podsakoff et al. (2009), factors associated with OCBO include: *Conscientiousness –* that refers to employees’ acceptance and adherence to the rules and regulations of the organization; *Sportsmanship –* that refers to a willingness to tolerate less than ideal circumstances; *Civic Virtue –* that refers to employees taking an active interest in the life of the organization. OCBOs have been referred to as generalized compliance (Organ and Konovsky, 1989) and are viewed as behaviors that occur because of expected rewards or the avoidance of punishment (Williams and Anderson, 1991).

Behaviors associated with OCBOs are considered to be more aligned with behaviors that are more consistent with people whose cognitive styles are more analytic than intuitive. For example, analytical people are known to have a more impersonal nature compared with the more interpersonal nature of intuitive people. Their focus within organizations tends to be toward initiating a higher proportion of task-oriented acts compared with intuitive people who prefer to engage in more socio-emotional oriented behaviors (Armstrong and Priola, 2001; Priola et al., 2004). Analytic individuals also show greater skills in cognitive analysis with a focus on detail (Pascual-Leone, 1989) and tend to be more compliant, adhering to company rules and regulations (Kirton, 1976). Erdil and Tanova (2015) also observed that analytic people tend to become more rule oriented and dependent on formal procedures. Such behaviors are consistent with the OCBO definition of conscientiousness (Podsakoff et al., 2009). According to Pascual-Leone (1989) analytic individuals are also more concerned about self-related benefits such as rewards and promotions than maintaining personal relationships. In the interest of generating self-related benefits, we would suggest that analytic people will therefore be more likely to tolerate less than ideal circumstances in their work endeavors (e.g., sportsmanship) and will be more inclined to take an active interest in the organization by, for example, attending functions that are considered important even though they may not be mandatory (e.g., C*ivic virtue*).

Again, on the basis that congruence of cognitive styles between leaders and their subordinates have revealed consistent and lasting positive effects for both dyadic partners (Deluga, 1998) including higher levels of communication satisfaction (Erdil and Tanova, 2015), and that deep-level similarities within vertical dyads will lead to partners being more committed and satisfied with their job experiences (Liao et al., 2008), we would further hypothesize that:

*H*_3_: Leader-follower congruence at the extreme analytic end of the cognitive style continuum (analytic follower-analytic leader) will lead to higher levels of follower OCBOs being reported by their leaders.

## Materials and Methods

### Sample and Procedure

We analyzed data from 125 leaders and 430 followers from six manufacturing organizations in the Peoples’ Republic of China. To limit common method bias (Podsakoff et al., 2012) we measured cognitive style based on leaders’ and followers’ self-ratings at time T1. At time T2 (1-week later) we measured leaders’ evaluations of followers’ OCB. Participation in the research was voluntary and confidentiality was guaranteed. The average tenure of participants was 9.14 years and their average age was 36. In terms of gender, 41.2% of participants were female. To control for common method bias, we followed the procedure suggested by Podsakoff et al. (2012) to measure independent and dependent variables from different sources. Data concerning follower OCB were collected from leaders’ rating. Both leaders and followers cognitive styles were measured using self-ratings.

### Measures

Because the original version of the research instruments were designed in English and the native language of the participants was Chinese, all questionnaires were translated using a back-translation procedure (Brislin, 1980). Two professional translators, fluent in both Chinese and English, were independently assigned to work on the translation process. The first of these translated all research instruments from English to Chinese. The Chinese versions were then sent to the second translator for translation back into English. Both original and translated English versions were then compared to identify any inconsistencies. Any differences were discussed between the researchers and both translators to determine any further revisions. Changes were minimal, meaning that we had achieved translation equivalence (Douglas and Craig, 1983).

#### Cognitive Style

We used the CSI to assess the analytic-intuitive dimension of cognitive style. The CSI (Allinson and Hayes, 1996) is a self-report questionnaire comprising 38 items, each comprising a true-uncertain-false response mode. Scores of 0, 1, or 2 are assigned to each response [Sample items: *In my experience, rational thought is the only realistic basis for making decisions (Analytic); I prefer chaotic action to orderly inaction (Intuitive)*]. The nearer the total score (38 items) is to the theoretical maximum of 76, the more analytic the respondent. The nearer the total score is to the theoretical minimum of 0, the more intuitive the respondent. Whilst the CSI represents a continuum, five notional styles associated with the CSI scores were defined as the 20th, 40th, 60th, and 80th percentiles in the distribution obtained from a sample of 1180 managers and professionals (Allinson and Hayes, 2015). Those are: *Intuitive* (score range 0–28); *Moderate Intuitive* (29–38); *Adaptive* (39–45); *Moderate Analytic* (46–52); and *Analytic* (53–76). Reliability of the CSI is excellent with a median Cronbach alpha coefficient (taken across 100 previous studies) being 0.84, and test-retest reliabilities ranging from 0.78 to 0.90 (Qi, 2011). Internal consistency reliability estimate for the present study was also 0.84. Construct validity is indicated by items loading on a single factor in many previous studies and significant correlations with various personality dimensions, national culture, and job level (Armstrong et al., 2002). Confirmation of its uni-factorial structure was recently reported by Armstrong and Qi (2016).

#### Organizational Citizenship Behavior

We used the 24-item OCB scale developed by Podsakoff et al’s (1990). Items were measured on a seven-point Likert scale ranging from (1) “Strongly Disagree” to (7) “Strongly Agree.” The scale comprised the five factors of conscientiousness, sportsmanship, and civic virtue (OCBO), and courtesy and altruism (OCBI) hypothesized by Organ (1988). Sample items from the sub-scales of OCBO and OCBI respectively were: *Obeys company rules and regulations even when no one is watching; is always ready to lend a helping hand to those around her/him*. Podsakoff et al’s (1990) study revealed internal consistency reliabilities of all five subscales that exceeded 0.80 and evidenced an adequate level of discriminant validity. Cronbach alpha values for the present study were as follows: overall OCB, α = 0.95; OCBO, α = 0.95; OCBI, α = 0.90.

## Data Analysis

First of all, one-way analysis of variance (ANOVA) was conducted to test whether there were differences across the six organizations on the tested variables. Results revealed that there were no significant differences for either OCB [*F*_(5, 409)_ = 1.78, *p* > 0.05] or cognitive style [*F*_(5, 409)_ = 2.05, *p* > 0.05]. It was therefore unnecessary to consider organization as a control variable in our analyses.

### Measurement of Congruence

The obvious way to assess congruence is to calculate the differences between leaders and followers CSI scores (Edwards and Parry, 1993). However, whilst difference scores have been widely used in organizational research (Edwards and Parry, 1993), this method is known to suffer from numerous methodological problems in the areas of reliability, spurious correlations and variance restriction (Edwards, 2001). We therefore used polynomial regression with response surface analysis to more precisely examine the exact nature and extent to which congruence between our predictor variables relate to our outcome variable (Edwards, 2009). This allowed us to analyze 3-dimensional surfaces relating to our congruence of cognitive style hypotheses, facilitated a clearer interpretation of results, and allowed us to see the effects of each of the component measures- leaders’ cognitive styles (LCS) and followers’ cognitive styles (FCS) on the outcome variables (OCB; OCBI; OCBO).

We followed Shanock et al. (2010) procedure for centering the predictor variables (LCS and FCS) about the midpoint of their respective scales. Then we created three new variables: (1) the square of the centered FCS variable; (2) the cross-product of the centered FCS and LCS variables; and (3) the square of the centered LCS variable (Table 2). Next, we ran the polynomial regression analyses. Results of the polynomial regressions were evaluated with regard to the four surface test valuesa_1_, a_2_, a_3_, and a_4_ (refer to Table 2). The slope of the line of perfect agreement (LCS = FCS) as related to overall OCB, OCBI, and OCBO is given by a_1_. Curvature along the line of perfect agreement as related to overall OCB, OCBI, and OCBO is given by a_2_. The slope of the line of incongruence (LCS = −FCS) is given by a_3_. The curvature of the line of incongruence as related to overall OCB, OCBI, and OCBO, indicating the degree of discrepancy between LCS, FCS, and the outcome variable is given by a_4_.

## Results

**FIGURE 1:**
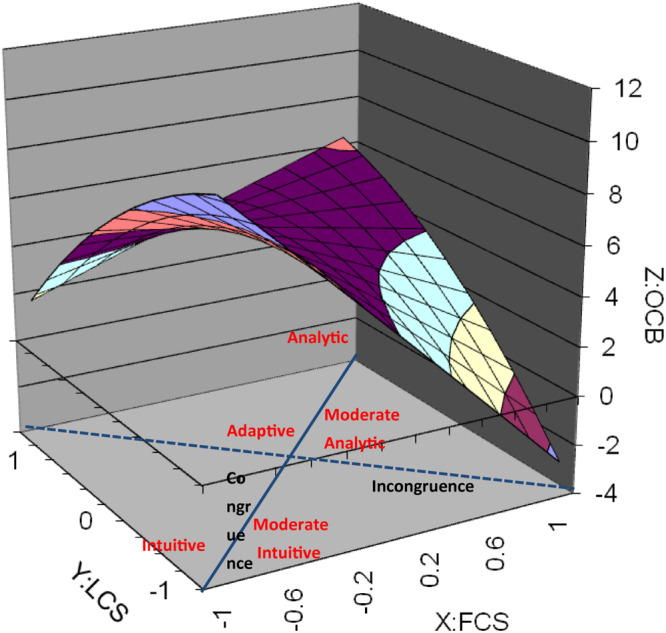
Response surface graph of overall OCB.

**FIGURE 2:**
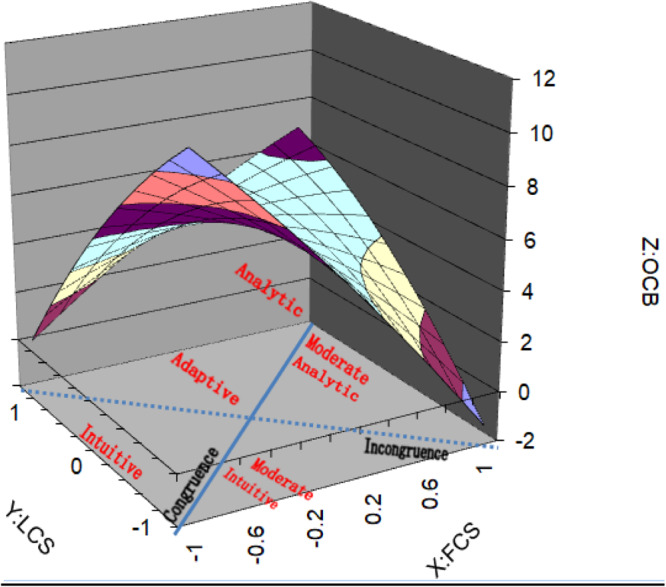
Response surface graph of OCBI.

**FIGURE 3:**
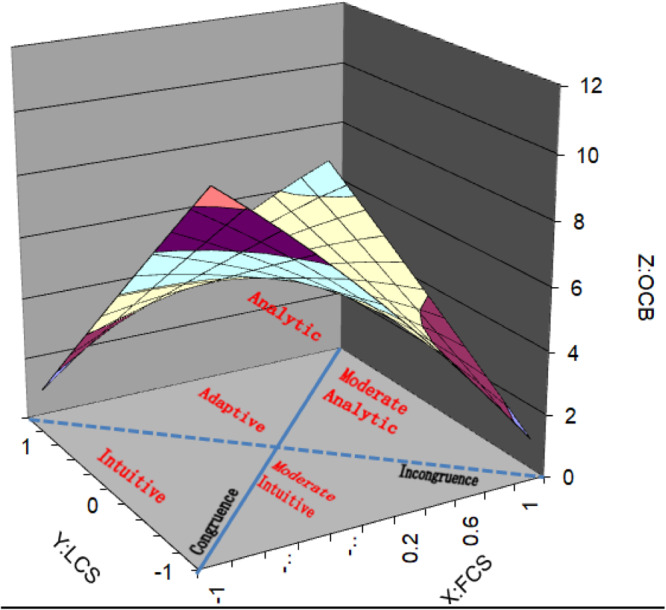
Response surface graph of OCBO.

Descriptive statistics for means, standard deviations, and correlations among variables are shown in Table 1. To aid interpretation of the results, three-dimensional response surface graphs have been produced (see Figures 1–3). From the graphs it should be noted that the X and the Y axes represent our predictor variables (FCS and LCS respectively), whereas the *Z*-axis represents our outcome variables, follower OCBI, OCBO, and overall OCB. A value of + 1 on both the X (FCS) and Y (LCS) axes represents an extreme preference for *Analysis* using logical and linear processing with a focus on detail. A value of −1 on the X and Y axes represents an extreme preference for *Intuition* using synthesis and simultaneous processing with a focus on assessment of the whole.

**TABLE 1:** Means, standard deviations, and correlations among variables.

**Variable**	***SD***	**Mean**	**1**	**2**	**3**	**4**	**5**	**6**	**7**	**8**
1. Employee age	9.12	36.34								
2. Educational background	3.87	4.36	0.10*							
3. Employee tenure	9.69	9.14	0.65**	0.09						
4. Follower CS	0.24	1.32	0.01	0.03	–0.01	(0.84)				
5. Leader CS	0.20	1.33	–	–	–	0.03	(0.84)			
6. OCB	0.99	5.21	–0.08	–0.02	0.04	−0.12*	0.14*			
7. OCBI	1.09	5.27	–0.07	0.01	0.06	−0.03	0.01	0.78**		
8. OCBO	0.93	5.16	−0.11*	−0.03*	0.03	−0.01	0.01	0.80**	0.77**	

Cronbach’s alphas are provided in parentheses on the diagonal. FCS, followers’ cognitive styles; LCS, leaders’ cognitive styles; OCB, organizational citizenship behavior. *p < 0.05, **p< 0.01.

Using these graphs we will firstly seek to determine whether congruence of follower cognitive style and leader cognitive style relate to overall OCB, OCBI, and OCBO. Secondly, we will analyze how the degree of discrepancy between follower cognitive style and leader cognitive style relate to overall OCB, OCBI, and OCBO. Thirdly, we will determine how the direction of the discrepancy between follower cognitive style and leader cognitive style relates to overall OCB, OCBI, and OCBO.

With regard to whether congruence of cognitive style relates to overall OCB, OCBI, and OCBO, the line of perfect agreement (congruence) is represented by the solid line between the front corners and the back corners of the graphs. As explained by Shanock et al. (2010), a linear relationship along this line as it relates to OCB, OCBI, and OCBO is indicated by variables a_1_ being significant and a_2_ being non-significant. If a_1_ is positive, OCB increases as both LCS and FCS increase. As shown in Table 2 (OCB), Table 3 (OCBI), and Table 4 (OCBO), the surface tests resulted in both a_1_and a_2_ being significant. This indicates a non-linear relationship along the line of perfect agreement as it relates to overall OCB, OCBI and OCBO. Since a_1_ is negative in all three cases (OCB:a_1_ = −2.04, *p* = 0.04: OCBI:a_1_ = −2.34, *p* = 0.04; OCBO:a_1_ = −2.05, *p* = 0.04), overall OCB, OCBI and OCBO decreases as both LCS and FCS increase (i.e., become more analytic). In Figures 1–3, the highest level of OCB, OCBI, and OCBO are at the front corners of the graphs where LCS and FCS are both low (more intuitive), and lower at the back corners of the graphs where LCS and FCS are higher (more analytic). An exception occurs when the extreme points of analysis are reached at the back corner where there is a small increase in OCB, OCBI, and OCBO. Since a_2_ is positive in all three cases (OCB:a_2_ = 3.00, *p* = 0.06: OCBI:a_2_ = 3.42, *p* = 0.04; OCBO:a_2_ = 3.30, *p* = 0.03), this suggests that the line of perfect agreement as it relates to OCB, OCBI, and OCBO is positive and a convex surface (upward curving) indicating that OCB, OCBI, and OCBO can increase more sharply as both LCS and FCS become lower or higher from some point.

**TABLE 2:** The relationship between cognitive similarity in leader-follower dyad and followers’ overall OCB.

**Variable name**		**Unstandardized betas**	**Standard errors**	**Covariances**
**Data entry area**				

	Constant	5.34		0.158
FCS	X (b1)	–2.05	0.579	0.024
LCS	Y (b2)	0.009	0.638	0.015
	X^2^ (b3)	–0.065	0.533	0.04
	XY (b4)	4.402	1.164	
	Y^2^ (b5)	–1.334	0.772	

**Effect**	**Coefficient**	**Standard error**	**Test stat (t)**	***p*-value**

**Testing slopes and curves**				

a1: Slope along x = y (as related to Z)	-2.04	1.03	–1.984	0.04
a2: Curvature along x = y (as related to Z)	3.00	1.55	1.941	0.06
a3: Slope along x = −y (as related to Z)	-2.04	0.65	–3.158	0.00
a4: Curvature along x = −y (as related to Z)	-5.80	1.50	–3.879	0.00

**TABLE 3:** The relationship between cognitive similarity in leader-follower dyad and followers’ OCBI.

**Variable name**		**Unstandardized betas**	**Standard errors**	**Covariances**
**Data entry area**				

	Constant	5.72		0.19
FCS	X (b1)	–1.44	0.64	0.03
LCS	Y (b2)	–0.90	0.70	0.02
	X^2^ (b3)	–0.49	0.59	0.05
	XY (b4)	4.91	1.28	
	Y^2^ (b5)	–1.01	0.85	

**Effect**	**Coefficient**	**Standard error**	**Test stat (t)**	***p*-value**

**Testing slopes and curves**				

a1: Slope along x = y (as related to Z)	−2.34	1.13	–2.08	0.04
a2: Curvature along x = y (as related to Z)	3.42	1.72	2.01	0.04
a3: Slope along x = −y (as related to Z)	−0.54	0.72	–0.75	0.46
a4: Curvature along x = −y (as related to Z)	−6.40	1.65	–3.88	0.00

**TABLE 4:** The relationship between cognitive similarity in leader-follower dyad and followers’ OCBO.

**Variable name**		**Unstandardized betas**	**Standard errors**	**Covariances**
**Data entry area**				

	Constant	5.51		0.14
FCS	X (b1)	–0.90	0.55	0.02
LCS	Y (b2)	–1.15	0.61	0.02
	X^2^ (b3)	–0.50	0.50	0.03
	XY (b4)	3.86	1.11	
	Y^2^ (b5)	–0.07	0.74	

**Effect**	**Coefficient**	**Standard error**	**Test stat (t)**	***p*-value**

**Testing slopes and curves**				

a1: Slope along x = y (as related to Z)	−2.05	0.98	–2.09	0.04
a2: Curvature along x = y (as related to Z)	3.30	1.47	2.24	0.03
a3: Slope along x = −y (as related to Z)	0.25	0.63	0.40	0.69
a4: Curvature along x = −y (as related to Z)	−4.43	1.42	–3.12	0.00

To interpret how the degree of discrepancy between LCS and FCS relates to OCB, OCBI, and OCBO we need to assess the curvature of the line of incongruence (LCS = −FCS) as it relates to OCB with a_4_ (OCB:a_4_ = −5.80, *p* = 0.00; OCBI:a_4_ = −6.40, *p* = 0.00; OCBO:a_4_ = −4.43, *p* = 0.00). The line of incongruence is represented by the dotted line between the left corner and the right corner of the graphs (Figures 1–3). A significant negative a_4_ indicates a concave surface whereby OCB, OCBI, and OCBO decreases more sharply as the degree of incongruence between LCS and FCS increases. This is shown on the graphs in Figures 1–3 where it is indicated that as LCS and FCS become more dissimilar, OCB, OCBI, and OCBO decrease sharply. These results demonstrate support for hypothesis 1.

Finally, determining how the direction of discrepancy between leader and follower cognitive styles is related to our outcome variable (indicated by the slope of the line of incongruence (X = -Y) as it relates to OCB) is assessed by considering variable a_3_. Table 2 (OCB), Table 3 (OCBI), reveal a a_3_ indicating a negative curvature along the line of incongruence as related to OCB. Figures 1–3 depict these results indicating that OCB, OCBI, and OCBO respectively are higher when the discrepancy between LCS and FCS are low. As the level of diversity between leader and follower increases such that LCS is higher than FCS and vice versa, OCB, OCBI, and OCBO decrease sharply. This shows that either side of the center of the graph, along the line of incongruence, OCB, OCBI, and OCBO decrease similarly as the discrepancy between FCS and LCS increases in either direction. This lends further support for hypothesis 1. From Figure 2, it can be seen that the highest level of OCBI occurs for the condition where both leader and follower are highly intuitive, lending support to hypothesis 2. Figure 3 reveals that whilst OCBO for the analytic dyad condition increases from the conditions of moderately analytic dyads, and adaptive dyads, this does not reach the level of OCBO for the condition where both leader and follower are both highly intuitive. There is therefore only partial support for hypothesis 3.

## Discussion

As hypothesized, results of our study are generally consistent with Byrne’s (1971) similarity attraction paradigm and suggest that congruence of follower and leader cognitive style is a predictor of follower OCB. With regard to incongruence and how the degree of discrepancy between leader cognitive style and follower cognitive style relates to OCB, our results reveal that OCB, OCBI, and OCBO are all highest for adaptive dyads where both leader and follower cognitive styles are in the center range of the cognitive style continuum. This is the point at which diversity of cognitive styles is lowest. Considering the line of incongruence in Figures 1–3, it is clear that OCB, OCBI, and OCBO all decrease sharply with increases in the degree of diversity between leader and follower cognitive styles. The lowest level of OCB occurs in situations where intuitive leaders are working with analytic followers. Intuitive leaders who adopt a global approach to processing information and feel comfortable acting and paying attention on the basis of gut feelings and hunches will see the behaviors of their analytic followers in sharp contrast to their own as those followers adopt more systematic approaches to investigation (Allinson and Hayes, 2015), thrive on attention to detail, and adopt step-by step approaches to processing information (Armstrong, 2000). Intuitive leaders may therefore have a relative intolerance for analytic followers and judge OCB more harshly. Conversely, analytic followers working with intuitive leaders may wonder “where on earth is this leading”?

Moving along the incongruence continuum (Figures 1–3) from the right hand corner (intuitive leader-analytic follower) to the left hand corner of the graphs (analytic leader-intuitive follower), OCB, OCBI, and OCBO are seen to decrease sharply again, although not to the same level as intuitive leader-analytic follower dyads. This difference may be due to analytic leaders being more tolerant of their intuitive followers, placing value on their ability to see links between unrelated ideas and experiences and to continually pursue new ideas and different approaches to decision making and problem solving.

Referring to the congruence continuum of Figures 1–3, our results revealed that the highest level of follower OCB, OCBI, and OCBO occurs in dyads where both leader and follower are intuitive, which represents a rather unique contribution. Those with intuitive cognitive styles are more divergent in their thinking and continually pursue new ideas and different approaches to problem solving and decision making. It is conceivable therefore that those intuitive leaders see their intuitive followers in good ways and enjoy high quality social and informational exchanges with them, leading to increased benefits on the part of the follower and reciprocal behaviors that benefit the organization.

It is also known that intuitive individuals exhibit a strong interest in people, preferring to help and maintain positive interpersonal relationships and are more inclined to shift their opinions to resolve conflicts (Armstrong et al., 2002). These behaviors are reminiscent of the courtesy and altruism factors of the OCB construct. When there is similarity between leaders and their subordinates, this has revealed consistent and lasting positive effects on supervisor related performance, and the promotion opportunities of subordinates (Deluga, 1998). Communication satisfaction between leaders and their subordinates is also known to be higher when leaders and their subordinates are both intuitive (Erdil and Tanova, 2015), further reinforcing higher quality leader-subordinate relations (Suazo et al., 2008) and the likelihood of increased OCBs.

A further consideration is that since our outcome variable is based on leaders’ perception, intuitive leaders may be better at judging OCB, seeing it in more subjective rather than objective terms which would be favored by analytic leaders. It is also noteworthy that intuitive information processors tend to place a greater emphasis on feelings (Armstrong et al., 2012), are generally more nurturing (Allinson et al., 2001) and adopt an interpersonal approach to problem solving (Armstrong et al., 2012). According to Suazo et al. (2008) these qualities associated with leader-follower relationships may be powerful mechanisms through which similarity influences followers’ positive state of psychological contract. Moving further along the congruence continuum (Figures 1–3) from intuitive dyads, through moderately intuitive dyads, adaptive dyads, and moderate analytic dyads, overall OCB, OCBI, and OCBO decline slightly, and then increases again for analytic dyads. However, this increase does not reach the same level as for congruent intuitive dyads. This difference may be due to analytics’ tendencies to focus more on tasks and goals rather than people, and to not valuing interpersonal relationships and human aspects as much as intuitive people. Analytic leaders are also likely to place more emphasis on logical thinking and therefore judge followers OCB, OCBI, and OCBO in more objective terms than intuitive leaders.

Finally, our study has also demonstrated the benefits of using a sophisticated statistical approach involving polynomial regression with response surface analysis in multi-source feedback research (e.g., leader-follower discrepancy). This has allowed us to examine the extent to which an outcome variable (OCB) is predicted by the two predictor variables (leader and follower cognitive styles) where the difference between these two variables is a central consideration. This approach is significantly more powerful and informative than using difference scores (absolute, algebraic or squared differences between two component measures) for analyzing discrepancies in ratings. Even though difference scores have been widely used in organizational research for studying congruence, methodological problems with using this approach are well known (e.g., Edwards and Parry, 1993; Edwards, 1994, 1995). For example, combining two distinct measures into a single score confounds the effects and contribution of each component measure on the outcome variable (Edwards, 2001). Using polynomial regression allows the effects of each component measure to be retained, making it possible to examine the contribution of each component measure to outcome variance. Additionally, using response surface methodology corresponding to the polynomial regression equations allows us to rigorously evaluate three-dimensional surfaces relating the component measures to outcomes (Edwards, 2009). The present authors would encourage further use of polynomial regression and response surface methodology in future diversity studies associated with evaluating the role of congruence. An excellent resource for helping with the application of these methods is Shanock et al. (2010).

## Implications

Notwithstanding the finer details of the preceding analyses, our over-arching arguments for congruence hypotheses related to leader-follower cognitive styles were largely upheld. This raises important considerations and implications. Firstly, results of the present study indicate that organizations may benefit from considering issues of similarity in their attempts to develop effective leader-follower partnerships and teams. Cognitive style is clearly an important basis for matching followers and leaders in order to increase followers OCB’s and this, in turn, is likely to lead to improvements in both individual and organizational success. Another fertile area of investigation for future congruence studies of this nature would be formal mentoring systems, building for example on the earlier work of Armstrong et al. (2002).

Secondly, some authors believe that cognitive strategies may be adopted to deal with a situation or perform particular tasks in the short term (Kirton, 1989). In this case, it may be possible through training for leaders to learn flexibility of style and adopt different approaches according to the styles of the followers with whom they are dealing in the interest of increasing followers OCB. To the authors’ knowledge there has been no previous work in this area.

Thirdly, awareness of cognitive styles through training and development are also useful for developing effective working relationships because a poor understanding of others’ styles can lead to frustration, disengagement or conflict. Intuitive members tend to get frustrated by analytic members’ insistence on analyzing every aspect of a situation and spending too much time gathering facts and pondering over information before coming to a decision. Conversely, analysts may find intuitive approaches to tasks frustrating and chaotic and often remain unconvinced by their arguments because of a lack of facts and logical arguments to underpin their reasoning.

Finally, our findings have demonstrated that the degree of match between leaders’ and followers’ cognitive styles may account for improved OCBs that are known to have a sizeable impact on the success and welfare of both organizations and individuals (Podsakoff et al., 2014; Chin, 2015). Organizations may therefore benefit from considering cognitive style theory as one criterion against which they may base their recruitment and selection criteria when choosing staff to work with particular leaders.

## Data Availability Statement

The datasets generated for this study are available on request to the corresponding author.

## Ethics Statement

Ethical review and approval was not required for the study on human participants in accordance with the local legislation and institutional requirements. Written informed consent from the participants was not required to participate in this study in accordance with the national legislation and the institutional requirements.

## Author Contributions

SA and MQ were all instrumental in the development of the research project, made significant contributions to the overall writing, and theoretical development of the manuscript. MQ carried out the data collection and ran the statistical analyses.

## Conflict of Interest

The authors declare that the research was conducted in the absence of any commercial or financial relationships that could be construed as a potential conflict of interest.
